# About Cocaine

**Published:** 1886-07

**Authors:** C. E. Francis

**Affiliations:** N. Y.


### ABOUT COCAINE.

BY DR. C. E. FRANCIS, N. Y.

At a recent meeting of the New York Odontological Society, the subject selected for discussion was "Cocaine and its Effects." Professor Doremus, of New York, the distinguished analytic chemist, was present, in response to a previous invitation, and addressed the society, giving his views concerning the alkaloid. Personally, his experience with this drug had been exceedingly limited, but he had heard of ill results following its use, and had then in his possession letters from two or three physicians who reported cases of partial paralysis which were *supposed* to have resulted from cocaine applied to aching or sensitive teeth. In one case, a woman had procured from a druggist a vial of tooth-ache drops containing cocaine, which she applied a number of times to quiet her aching tooth, and afterwards experienced "a peculiar feeling of numbness, that might have resulted in death had not a physician been called in;" at least so wrote the attending physician to Prof. Doremus. The second case reported by letter to the Professor had reference to the personal experience of Dr. Hoyt, a physician of New York, who, it was alleged, had been "a victim of cocaine poisoning." Dr. Hoyt being present at the meeting, responded to a request of the president by stating his case, which in substance was as follows: In his mouth was an inferior incisor which needed filling. He called on a city dentist, who, judging from the doctor's remarks, was a gentleman of intelligence and of excellent professional record, in order that he might receive the needed attention. In preparing the cavity for a filling the dentine was so exquisitely sensitive that little progress could be made, so in order to render the operation endurable, a small quantity of a four per cent. solution of muriate of cocaine was applied to the cavity on a pellet of cotton. This, the doctor "imagined," slightly modified the pain for the instant, but as the operation of excavating was renewed the pain was as keen as ever, so more cocaine was applied, and this repeated at intervals five or six times, but with no marked effect as a pain obtunder. The rubber dam was finally adjusted and the cavity filled with gold, but the gold was not securely anchored, so it became dislodged by the instrument used in trimming away the surplus material around the cavity margin. The tooth was then filled with oxy-phosphate of zinc. No special trouble was experienced until next morning, when the doctor had a feeling of numbness on that side of his face, and had considerable difficulty in swallowing his breakfast. His right arm also became benumbed, suggesting something akin to paralysis. This condition continued two days, then gradually got better, but it was several weeks before he was quite well again.

From the Doctor's remarks it did not seem clear to some of the audience whether or not the pulp was exposed when the cocaine was applied. The Doctor did not seem positive, but he thought it "not quite, but nearly exposed." Dr. Hoyt was asked if he had any theory that would explain his condition, or if he believed it due to the action of cocaine applied to his tooth? In reply the Doctor said that the intense pain he suffered during the operation may have affected the nerves of his face, and the application of cocaine may have had a specific action on the nerve in the tooth which also affected the facial nerves—or that the operation and the cocaine together had done the business.

Although cocaine has been employed within the past two years in thousands upon thousands of instances by oculists, dentists, surgeons and physicians in general practice in this and other countries, and has also been dispensed by druggists in all communities for various troubles of a painful character, very few cases have been reported where its use is supposed to have produced serious ill effects, and even in the few cases reported the diagnosis of each has been exceedingly vague and unsatisfactory. Cocaine has been dropped into the eye in innumerable instances for operations on that organ. It has found its way into the nares and in every part of the oral cavity. It has played an active part in the removal of tumors, and for dressing burns and wounds of every character has done great service. In cases of neuralgia it has been applied in form of a lotion, also injected hypodermically. It has been largely used as a tonic, and in countries where the coca leaves are produced the natives have for many years been in the habit of chewing them, or imbibing a decoction made therefrom. And yet what agent of like potency, so freely used, has been the cause of less mortality? Indeed, have any well authenticated cases of death by "coca poisoning" been reported?

In our specialty the use of cocaine is considerably limited, as it affects only soft tissue. Applied to sensitive dentine it is valueless, and to inject hypodermically with a view to render dental operations less painful is a practice of doubtful expediency. It, however, has its place as a local anæsthetic, and is valuable for certain purposes, and therefore should not be too hastily condemned, or unreasonably so. That it may prove an agent of mischief if used indiscriminately can hardly be denied, and due caution is requisite in the employment of not only this, but all drugs possessing such marked characteristics.

